# The impact of CARA mandates on nurse practitioner controlled substance prescribing in Oregon: a cohort study

**DOI:** 10.1186/s13011-022-00431-z

**Published:** 2022-01-31

**Authors:** Tracy A. Klein, Daniel Hartung, Sheila Markwardt

**Affiliations:** 1 College of Nursing, Washington State University Vancouver, 14204 NE Salmon Creek Avenue, Vancouver, WA 98686-9600 USA; 2 OSU/OHSU College of Pharmacy, Oregon Health and Science University, Collaborative Life Sciences Building (CLSB), 2730 SW Moody Ave., CL5CP, Portland, OR 97201-5042 USA; 3 OHSU Biostatistics and Design Program, Oregon Health and Sciences University, 3181 SW Sam Jackson Park Road, Portland, OR 97239-3098 USA

**Keywords:** Buprenorphine, Nurse practitioner, Substance use disorder, Opioids

## Abstract

**Background:**

In 2017, the United States Comprehensive Addiction and Recovery Act (CARA) expanded authorization to prescribe buprenorphine for opioid use disorder (OUD) to nurse practitioners (NPs). Compared to physicians, NPs were required to complete 16 additional hours of training on controlled substance prescribing before a buprenorphine waiver application. As this differential additional education mandate was seen as a potential barrier, we evaluated the impact of this requirement on both NP waiver acquisition and prescribing of controlled substances, comparing NPs who obtained waivers to those who had not.

**Methods:**

Through 2016–2018 Oregon Prescription Drug Monitoring Program and linked NP licensure data, we identified factors associated with waiver acquisition at baseline (2016) and evaluated changes in controlled substance prescribing before (2016) and after waiver acquisition (2018). Using chi-square and Mann-Whitney U testing, we calculated and described controlled substance prescribing types, rates, and patient level quantities including co-prescribing of benzodiazepines and opioids by NPs. Multivariable linear regression compared prescribing by waivered and non-waivered NPs for significant changes in non-buprenorphine controlled substance prescribing.

**Results:**

Waivered NPs were more likely to have a psychiatric certification, have prior disciplinary action, and have generally higher levels of non-buprenorphine controlled substance prescribing than their non-waivered counterparts. While there was a significant increase in opioid prescriptions per patient among waivered NPs, following CARA implementation, co-prescribing of benzodiazepines and opioids significantly declined among waivered NPs relative to non-waivered NPs.

**Conclusions:**

Although educational requirements were rescinded in 2021 for most applicants, enhanced opioid prescribing training should be incorporated into professional educational offerings regardless of regulatory mandate. We recommended continued focus on education regarding avoidance of high risk prescribing such as co-prescribing of opioids and benzodiazepines. NPs who acquire waivers may take on higher risk patients already using opioids, and these findings may represent transitions in practice and patient setting.

## Background

United States federal law and regulation expanded access to buprenorphine for treatment of opioid use disorder (OUD). In 2016 Congress passed the Comprehensive Addiction and Recovery Act (CARA) which extended prescriptive authority for office-based treatment with buprenorphine to nurse practitioners (NPs) for up to 30 patients beginning in 2017 [1]. Prior studies demonstrate both a geographic maldistribution of providers and the potential contribution of NP prescribers to easing this disparity [2, 3]. Barriers to providing buprenorphine are similar for NPs and physicians, including concerns about medication diversion or misuse and lack of access to mental health backup and support [4]. Studies have shown NPs have increased buprenorphine access, especially in underserved areas [3].

Unlike physician assistants, who are required to practice with a supervising physician, NPs have autonomous practice in many states. As of December 2021, 24 states and the District of Columbia confer full scope of practice to NPs without a requirement for physician involvement in prescribing decisions [5]. States that are less restrictive of NP prescribing had larger increases in NP buprenorphine prescribing than states that are more restrictive [6, 7]. An expanded understanding of additional facilitators and barriers to buprenorphine prescribing in states which provide little regulatory constraint is critical to continued assessment of federal law efficacy and success.

NPs are granted full prescriptive authority for scheduled drugs in the state of Oregon, which was among states with the largest increase in NP prescribing of buprenorphine, particularly in very rural areas after implementation [2]. NPs in Oregon may be designated as primary care providers and the family nurse practitioner is the most common license designation (Oregon State Board of Nursing, personal communication, March 8, 2019). Broad controlled substance authority has been in place since the 1990’s, facilitating analysis of long term autonomous prescribing patterns.

In late 2020, the Trump administration promulgated rule changes to remove the DEA waiver requirement for physicians who prescribe buprenorphine to fewer than 30 patients [8]. Although the incoming Biden administration delayed advancement of those changes, they were reinstated with an expanded scope in April 2021. Specifically, the rule changes stipulate that prior educational requirements to obtain a waiver to prescribe buprenorphine will no longer be mandated, but that notification of intent to prescribe will still be required [9]. Professional recommendations continue to support non-regulatory integration as an academic expectation for health professionals [10, 11]. This may be particularly relevant for NPs, who were required to complete 16 additional post licensure training hours to become authorized to prescribe buprenorphine as compared to physicians [12]. Previously required modules offered to NPs included content on assessment protocols, treatment of elderly and pregnant patients, avoidance of stigmatizing language, and the roles, responsibilities, and limitations of practice with buprenorphine. A module specifically focusing on management of other substance use disorders (benzodiazepines, cocaine, stimulants and cannabis) was also required [12].

The effect of these additional educational requirements on non-buprenorphine controlled substance prescribing has not been evaluated. It is critical to examine how and if expanded educational requirements impacted prescribing of controlled substances overall. While OUD is the target of buprenorphine therapy, prescribers authorized to provide it are not restricted from continued prescribing of opioids or other controlled substances such as benzodiazepines which can create risk for patients, and a history of substance abuse diagnosis is associated with high dose benzodiazepine prescribing by primary care providers [13].

Characteristics of NPs who prescribe buprenorphine and where they practice likely differ from those who prescribe controlled substances generally. County level variables which facilitate buprenorphine prescribing include treatment capacity, overdose rate, and percentage of male non-Hispanic white population [6]. Prescriber variables which are publicly available in addition to licensing demographic characteristics include disciplinary status and history. Because nurses are revoked and fined at a higher rate than physicians and also have a higher rate of probation for their own substance use disorder offenses, inclusion of disciplinary data was explored in this study as a unique characteristic which may impact prescriptive authority and controlled substance practice [14].

The objectives of this study were: 1) to describe characteristics of NPs who obtained a waiver in Oregon to prescribe buprenorphine (waivered) compared to those who did not (non-waivered) and 2) to evaluate changes in non-buprenorphine controlled substance prescribing following CARA implementation between waivered and non-waivered NPs. We hypothesize that increased educational components for waivered NPs would change controlled substance prescribing as measured by key variables associated with patient use and dispensing patterns.

## Study data and methods

### Data source and sample

We excluded all non-NP prescribers from this study as well as any prescriptions for buprenorphine products (alone or in combination). We included all NPs with one or more prescriptions for either an opioid or benzodiazepine prescription dispensed by a pharmacy and entered into the Oregon Prescription Drug Monitoring Program (PDMP) database from January 1, 2016 to December 31, 2018 (*n* = 396,385) prescriptions. This dataset includes information on drug name, strength, quantity dispensed, days’ supply, date dispensed, and prescriber. NP characteristics were determined through a Drug Enforcement Agency (DEA) number linked demographic and licensure database from the Oregon Board of Nursing. The Board of Nursing licensure database contains demographic and practice information such as age, NP specialty certifications, licensure date, and prior professional disciplinary action.

To maintain confidentiality specified in the data use agreements, age (as of data request 3/18/2019) and licensure time was categorized into five-year increments by the Oregon Health Authority prior to data release. For professional certification, we identified if NPs had a psychiatric or mental health specialty certification. DEA waiver-status was provided by Oregon’s PDMP program.

### Methods and statistical analysis

Our first objective was to identify factors associated with NP waiver acquisition. We compared demographic and licensure characteristics between NP who ultimately received an waiver and those who did not. We also used PDMP dispensing data to compare controlled substance prescribing patterns between these two groups of NPs using 2016 (pre-CARA) as the baseline year. For each NP, we calculated the number of unique patients to whom they prescribed opioid analgesics, the average number of opioid prescriptions they prescribed to each patient, the number of patients to whom they prescribed long-term opioid therapy, and the number of patients who received at least one opioid prescription at or above 90 morphine milligram equivalents (MME) per day [15]. We considered patients with more than 90 days’ supply dispensed during the year as having long-term opioid therapy. We converted opioid doses to MMEs using standard CDC endorsed conversion factors [16]. We also determined the number of patients to whom each NP prescribed benzodiazepines and the average number of benzodiazepine prescriptions prescribed per patient. Finally, we examined the number of patients who were co-prescribed both an opioid and benzodiazepine, defined as overlapping days’ supply. Comparing waivered versus non-waivered NPs, we used chi-square tests for categorial data and Mann-Whitney U tests for continuous data.

Our second objective was to evaluate changes in controlled substance prescribing among waivered NPs relative to those who were not. Using the prescribing metrics described above, we used multivariable linear regression to compare changes in prescribing patterns in 2018 relative to 2016 (prior to CARA waiver acquisition) across groups. In each regression model, the dependent variable was operationalized as the within NP difference in each prescribing metric; independent variables included waiver status, age, years in practice, psychiatric specialty, prior discipline, and the prescribing metric at baseline (2016). *P*-values less than 0.05 were considered statistically significant. All analyses were performed using Stata/SE 15.1 (StataCorp. 2017. College Station, TX).

## Results

Of the 3321 NPs identified in the Board of Nursing licensure data, 187 were identified as acquiring a wavier by the Oregon Health Authority. Demographic and prescribing characteristics for these NPs are summarized in Table 1. Waivered nurse practitioners were significantly more likely to be psychiatric certified (29% vs 13%; *p* < 0.001) and have had prior discipline (5% vs 3%; *p* = 0.025) than non-waivered NPs.

**Table 1:** Demographic, professional, and 2016 prescribing characteristics of nurse practitioners

Characteristics	Waivered (***n*** = 187)	Non-Waivered (***n*** = 3134)	p-value^**1**^
Age group, count (%)			
Less than 35	21 (11.2%)	407 (13.0%)	0.79
35–49	77 (41.2%)	1319 (42.1%)	
50–64	69 (36.9%)	1055 (33.7%)	
65+	20 (10.7%)	353 (11.3%)	
Specialty: Psychiatric/Mental Health, count (%)	54 (28.9%)	412 (13.1%)	< 0.001
Years in practice, count (%)			
Less than 5	95 (50.8%)	1517 (48.4%)	0.27
5–9	41 (21.9%)	584 (18.6%)	
10–14	18 (9.6%)	346 (11.0%)	
15–19	9 (4.8%)	241 (7.7%)	
20–24	17 (9.1%)	237 (7.6%)	
25+	7 (3.7%)	209 (6.7%)	
Ever had prior discipline, count (%)	10 (5.3%)	81 (2.6%)	0.025
**2016 Controlled Substance Prescribing, count (%)**	**Waivered, 127 (67.9%)**	**Non-Waivered, 1323 (42.2%))**	< 0.001
Patients with an opioid prescription, mean (SD) [median (IRQ)]	92.7 (155.9) [31.0 (2.0, 125.0)]	72.0 (130.2) [25.0 (2.0, 98.0)]	0.34
Opioid prescriptions per patient, mean (SD) [median (IRQ)]^2^	2.8 (1.8) [2.4 (1.2, 3.8)]	2.2 (1.8) [1.3 (1.0, 2.6)]	< 0.001
Patients with long-term opioid therapy, mean (SD) [median (IRQ)]	25.0 (72.2) [2.0 (0.0, 30.0)]	13.1 (48.0) [0.0 (0.0, 6.0)]	< 0.001
Patients with a benzodiazepine prescription, mean (SD) [median (IRQ)]	43.3 (46.7) [31.0 (8.0, 62.0)]	28.2 (40.9) [10.0 (2.0, 39.0)]	< 0.001
Benzodiazepine prescriptions per patient, mean (SD) [median (IRQ)]^2^	2.8 (2.2) [2.3 (1.4, 3.7)]	2.4 (1.6) [1.7 (1.0, 3.3)]	0.003
Patients co-prescribed opioid and benzodiazepine, mean (SD) [median (IRQ)]	4.4 (7.1) [1.0 (0.0, 6.0)]	3.6 (10.2) [0.0 (0.0, 3.0)]	0.003

Abbreviations: *IQR* interquartile range, *MME* morphine milligram equivalents

^1^*p*-values are from chi-square test for categorical variables and Mann-Whitney U tests for continuous variables

^2^Conditional on patient having received prescription described

Of the NPs included, 1450 had one or more controlled substance prescription in Oregon’s PDMP in 2016. At baseline (2016 pre-CARA), waivered NPs were more likely to have prescribed controlled substances than non-waivered NPs (68% vs 42%; *p* < 0.001). Prior to waiver acquisition, waivered NPs wrote significantly more opioid prescriptions per patient than non-waivered NPs (mean 2.8 versus 2.2 prescriptions per patient; *p* < 0.001) and had more patients with long-term opioid therapy (mean 25.0 versus 13.1 patients; *p* < 0.001). NPs who became waivered also prescribed benzodiazepines to more patients (mean 43.3 versus 28.2; *p* < 0.001) and with higher intensity (2.8 vs 2.4 prescriptions per patient; =0.003) than those who did not. Waivered NPs co-prescribed opioids and benzodiazepines to more patients than those who were not (4.4 vs 3.6 patients; *p* = 0.003).

**Fig. 1:**
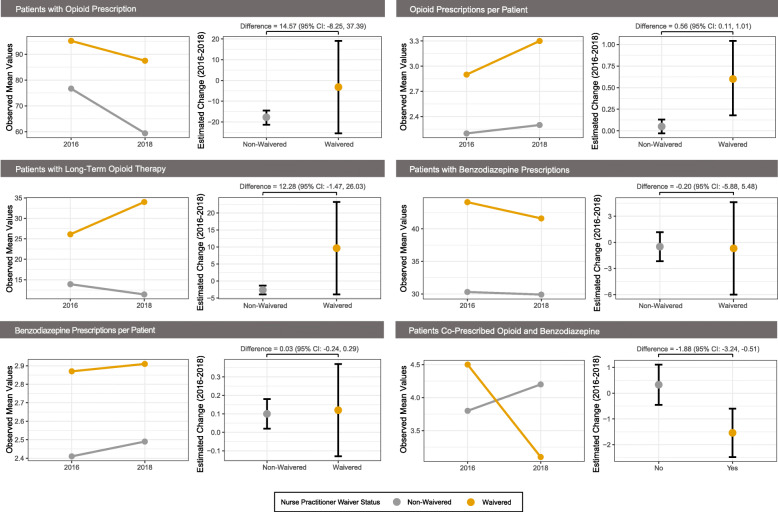
Differences between waivered and non-waivered NPs 2016-2018

Regression model estimates are summarized in Table 2. Figure 1 graphically depicts changes in controlled substance prescribing following CARA implementation among waivered and non-waivered NPs. Although the number of patients prescribed an opioid declined less for waivered NPs compared to non-waivered NPs, the differences between the two groups was not statistically significant. Similarly, the number of patients prescribed long-term opioid therapy decreased significantly for non-waivered NPs (− 2.64 patients; 95% CI − 3.95 to − 1.33) but remained statistically unchanged for waivered NPs; differences between waivered and non-waivered NPs were also non-significant. The number of opioid prescriptions per patient increased for waivered NPs compared to non-waivered NPs (0.56 prescriptions per patient: 95% CI 0.11 to 1.01). Although there were minimal changes in benzodiazepine prescribing overall, there was a significant decrease in co-prescribing of benzodiazepines and opioids by waivered NPs compared to non-waivered NPs (− 1.88 patients; 95% CI − 3.24 to − 0.51).

**Table 2:** Controlled substance prescribing change by waiver status

	***2018–2016 Difference (95% CI)***^***1***^		***Difference in Difference (95%CI)***^***23***^
***Model***	***Waivered***	***Non-Waivered***	
Patients with an opioid prescription	−3.19 (−25.48, 19.10)	**−17.76 (− 21.37, − 14.15)**	14.57 (−8.25, 37.39)
Opioid prescriptions per patient	**0.61 (0.18, 1.04)**	0.05 (−0.03, 0.13)	**0.56 (0.11, 1.01)**
Patients with long-term opioid therapy	9.64 (− 3.92, 23.20)	**−2.64 (− 3.95, − 1.33)**	12.28 (− 1.47, 26.03)
Patients with a benzodiazepine prescription	−0.69 (−6.00, 4.62)	−0.49 (− 2.16, 1.18)	−0.20 (−5.88, 5.48)
Benzodiazepine prescriptions per patient	0.12 (− 0.13, 0.37)	**0.10 (0.02, 0.18)**	0.03 (− 0.24, 0.29)
Patients co-prescribed opioid and benzodiazepine	**−1.54 (− 2.48, − 0.60)**	0.33 (− 0.45, 1.11)	**−1.88 (− 3.24, − 0.51)**

Abbreviations: *CI* confidence interval, *SE* standard error

^1^Model estimated difference in each outcome from 2016 to 2018 for waivered and non-waivered nurse practitioners; positive values indicate an increase and negative values indicate a decrease in each outcome

^2^Model estimated difference in difference. This is the difference between waivered and non-waivered nurse practitioners in the 2016 to 2018 difference of each outcome; values in bold are statistically significant at the 0.05 level

^3^Regression models were controlled for the following: baseline age, years in practice at baseline, psychiatric or mental health specialty, prior discipline at baseline, and baseline outcome value

### Limitations

Our study has limitations. Aggregate prescribing data does not capture patient characteristics, such as diagnoses and comorbidities, which affect prescribing decisions and practices. Confidentiality of both PDMP and licensure databases constrain linking and further exploration of individual patient and prescriber variables which may lend deeper understanding of drug use and rationale for prescribing. Prescribing can also be influenced by a number of external factors including insurance coverage, clinic level policies, and panel population, none of which are in the PDMP database. Prescriptions in the PDMP database lack additional prescriber characteristics such as the waiver acquisition date and the number of patients a prescriber may see. Finally, the study was restricted to NPs prescribing for patients in Oregon, which limits generalizability. No comprehensive national PDMP database currently exists, and data must be obtained and analyzed on a state-by-state basis.

## Discussion

In this study we found waivered NPs to be more likely to have a psychiatric certification, have prior disciplinary action, and have generally higher levels of controlled substance prescribing than their non-waivered counterparts. Following CARA implementation, co-prescribing of benzodiazepines and opioids significantly declined among waivered NPs relative to non-waivered NPs. There was also a significant increase in opioid prescriptions per patient among waivered NPs.

Similar to our findings, early waiver studies among physicians found adoption by psychiatric and addiction medicine specialties with subsequent diffusion to primary care [6]. Predictors of addiction specialist waiver acquisition and prescribing of buprenorphine included organizational support of buprenorphine training and use, more time spent in psychiatry or general group practice, seeing 10 or more opioid dependent patients in the last month, and the belief that prescription drugs play a large role in addiction treatment [17]. In 2018 NPs represented the greatest increase in buprenorphine prescribing rates by prescriber type nationally, while psychiatric and addiction medicine physicians decreased by − 8.8 and − 6.7% respectively [6]. While subspecialty certification (such as addiction management) was not provided for this population, waivered NPs were twice as likely to be psychiatric providers as non-waivered NPs. The early waiver adoption by this group may be attributable to a preexisting interest in treatment of addiction, the high comorbidity of psychiatric and addictive disorders needing comprehensive treatment approaches for their patient population, and confidence in individual nursing skill and expertise. As the authority of NPs to manage buprenorphine evolves, and federal and state policies continue to normalize treatment in primary care, it is likely that a similar decrease in specialty prescribing of buprenorphine nationally will be seen for NP prescribers as that which was noted by Roehler et al. for physician prescribers [6].

Waivered NPs had a small but statistically significant higher rate of history of discipline with their licensing board. This may represent.

NP discipline related to prescribing or personal substance use disorder, however such history often restricts expanded DEA privileges and is grounds for DEA privilege revocation [18]. Disciplinary sanctions unrelated to prescribing or substance use disorder are quite rare for NPs and may represent variance in patient risk level and setting [19]. We also found waivered NPs had higher baseline rates of prescribing benzodiazepines and opioids. Prescribing practices for controlled substances are influenced by a number of different factors including state laws, formularies, and state scopes of practice. Although limitations of the PDMP preclude further description of patient diagnoses or prescribing indications, it is likely that waivered NPs were already caring for patients with opioid use disorder or dependence.

Finally, we identified several notable changes in non-buprenorphine controlled substance prescribing among waivered NPs relative to non-waivered NPs. Consistent with larger population-wide opioid prescribing trends, non-waivered NPs had large declines in the number of patients who received an opioid prescription and who received long-term opioid therapy. In contrast, patients receiving opioids, or long-term opioid therapy, did not change significantly among waivered NPs. In fact, opioid prescribing intensity per patient increased significantly among waivered NPs. The reasons for these changes are not completely clear but possibly represent desired treatment goals as patients are shifted to providers who will manage their long-term use with alternatives including either opioid de-escalation and/or eventual transition to buprenorphine. The significant decrease in co-prescribed benzodiazepines and opioid constitutes an important reduction in a particularly risky practice that might reflect the additional prescribing education conferred through the waiver process [13].

In this study we examined characteristics of waivered NPs and evaluate the association between waiver acquisition and non-buprenorphine controlled substance prescribing. For NPs (and physician assistants), initial waiver acquisition required additional training above and beyond that which was required by physicians. Although continuing education can improve performance, its link to patient outcomes is tenuous [20]. While this educational requirement has now been eliminated, our data suggest it may have had utility for practitioners treating high-risk patients. Effective continuing educational methods are known more interactive, use diverse delivery methods, and involve multiple exposures rather than a one-time requirement [20]. The academic setting provides an opportunity to both engage in sustained content and practice reflective application of skills learned. We suggest that there be a transitional shift from mandated buprenorphine education linked to a DEA number to integration into health care professional academic training with standardized learning objectives and goals. Interprofessional substance use disorder educational interventions offer an opportunity to improve health professions students’ knowledge, skills, and attitudes toward SUDs and interprofessional collaboration [20]. Federal funding through the Substance Abuse and Mental Health Services Administration has been awarded to multiple universities to further the education and training of students in the medical, physician assistant, and nurse practitioner fields in order to increase numbers and access to prescribers for OUD [21].

However, additional support is needed to engage other healthcare professionals who might facilitate or limit buprenorphine access. For example, a recent study found 20% of pharmacies limited buprenorphine access for patients with OUD [22]. Inclusion of early education about OUD and its integration into routine health assessment, screening and treatment in the primary care setting can help normalize this practice and reduce differential barriers to access of this effective treatment.

## Conclusions

Our results found that waivered NPs significantly changed their practice regarding the high-risk co-prescribing of benzodiazepines and opioids as compared to non-waivered NPs. Given the removal of the educational mandate to become a waivered prescriber, consideration should be given to multiple methods of continued support and interprofessional education regarding not only safer provision of controlled substances but also effective ongoing treatment of substance use disorders.

## Data Availability

The datasets analyzed during the current study are available from the corresponding author on reasonable request and with prior permission of the Oregon Health Authority.

## Abbreviations

CARA: Comprehensive Addiction Recovery Act
DEA: Drug Enforcement Administration
NP: Nurse Practitioner
OUD: Opioid Use Disorder
PDMP: Prescription Drug Monitoring Program
